# Editorial: Advancing the treatment landscape in Parkinson's disease using sensor technology and data-driven modeling

**DOI:** 10.3389/fnagi.2022.1113818

**Published:** 2022-12-20

**Authors:** Ritesh A. Ramdhani, Anahita Khojandi, Brian H. Kopell

**Affiliations:** ^1^Department of Neurology, Donald and Barbara Zucker School of Medicine at Hofstra-Northwell, Hempstead, NY, United States; ^2^Department of Industrial and Systems Engineering, The University of Tennessee, Knoxville, Knoxville, TN, United States; ^3^Icahn School of Medicine at Mount Sinai, New York, NY, United States

**Keywords:** Parkinson's disease, sensors, machine learning, gait disorder, treatment

Effectively managing Parkinson's disease (PD) symptoms is a formidable challenge for healthcare providers. PD patients exhibit considerable clinical heterogeneity in their symptoms, yet many patients struggle with receiving specialized care to personalize their treatment strategies. Advanced PD is associated with emergence of motor complications (i.e., motor fluctuations and dyskinesia), axial symptoms including gait, postural and balance disorder as well as freezing of gait (FoG), and non-motor symptoms such as sleep disturbances and neurobehavioral disorders. Studies classifying Parkinson's clinical subtypes have predominantly relied on clinimetrics. Data driven approaches incorporating motor and non-motor symptoms associated with the disease have expanded the clinical spectrum with recent studies demonstrating overarching phenotypic clusters: mild motor predominant, non-motor predominant, intermediate, diffuse malignant (Fereshtehnejad et al., 2017; Mu et al., 2017). However, these phenotypic classifications and treatment response measurements remain confined to non-granular, subjective longitudinal assessments.

Sensor technology, which represents a quickly growing consumer market in the U.S. and globally, has emerged as a tool capable of providing objective motor performance data on individuals afflicted with PD– expanding the horizon of clinical care for this neurodegenerative condition. This includes an array of electromechanical wearable sensors as well as motion sensing input devices such as cellphone cameras, among others. One in five U.S. adults currently uses a wearable health tracker or “smart watch.” Also, ~80–90% of all mobile devices used globally are designated a smart-phone. Given their widespread use, such sensors are increasingly being utilized to monitor patient health states and behaviors. The articles published in the Research Topic: *Advancing the treatment landscape in Parkinson's disease using sensor technology and data-driven modeling*, present innovative methods that use sensor technology and advanced computing approaches to delineate complex symptoms (i.e., motor fluctuations, axial symptoms, and cognition) more precisely, enhance symptom monitoring, and gauge treatment response.

Axial symptoms have always been thought of and shown to be resistant to medications, thus, creating significant long-term disability for those afflicted. Two articles in this Research Topic sought to better delineate these symptoms. The first by Wu et al. utilized a motion capture device in 44 PD patients and 21 healthy controls to demonstrate that axial symptoms were indeed responsive, to a certain degree, to dopaminergic regimen. With more precise kinematic data, the researchers report improved time to rise from a chair, increased lower limb kinetics (i.e., stride length, gait velocity) and reduced truncal flexion in the PD cohort. In an article by Li et al., the authors proposed a novel multi-stage freezing of gait recognition model based on motion feature extraction of video using machine learning methods from a mobile phone video of timed up and go tests in 50 PD subjects. This model's recognition performance demonstrated good sensitivity and specificity–offering an opportunity to investigate its utility at a larger population level as FoG is one of the primary catalyst for falls and deterioration of quality of life in advanced Parkinson's patients. Recognizing FoG in a patient's native surroundings not only stands to enhance how it is managed but can influence future treatment trial design and execution.

Using objective, quantitative data to improve treatment planning for PD patients is another frontier that is currently being explored. Two articles in this Research Topic sought to leverage sensors and computing to characterize various aspects of the disease and symptoms in order to influence the treatment approach. Farzanehfar et al. used a wearable sensor [Parkinson's KinetiGraph (PKG^®^)] to identify patients experiencing “wearing off” periods, referred to as fluctuators, and showed that using objective and quantitative sensor data to guide treatment for these patients, as opposed to conventional non-sensor-based management strategies, provided better outcomes in terms of clinical scales. In another article by Pelosin et al., the authors used treadmill training with virtual reality (TT + VR) to examine the efficacy of various training plans in improving gait performance and cognitive function. The system included a camera (Kinect^®^) to collect quantitative measures of subjects' feet movement while walking on the treadmill and incorporated them into a computer-generated simulation along with virtual obstacles that are projected on a screen placed in front of the treadmill for subjects' view. The training included subjects walking on the treadmill while avoiding these virtual obstacles where they were assigned to either a 6- or a 12-week group (3 sessions/week). The results showed similar improvements of gait performance in both groups. However, the fall rate and fear of falling were decreased further in the 12-week group. Interestingly, improvements in cognitive functions were only seen in subjects after prolonged training (the 12-week group). Hence, objective, quantitative data has the potential to create new and improved treatment plans for PD patients while addressing a wide range of symptoms.

Finally, understanding the limitations of current sensor technology when it comes to PD and fine-tuning the methods to improve symptom monitoring is critical to move the field forward with respect to clinical diagnosis and management. One article in this Research Topic, by Shokouhi et al., sought to examine the accuracy of step count in PD using wearable sensors. A total of 21 normal walkers (10 without PD) and 27 abnormal walkers were videoed while wearing a sensor (PKG) and the data were analyzed. The study revealed that the accuracy of step count was high in normal walkers but not in abnormal walkers and using a measure of the loss of rhythmicity in walking could assist in detecting the likelihood of error in step count. In particular, the study showed that care must be taken when evaluating the step count results for subjects with bradykinesia due to the potentially higher likelihood of step count errors.

In summary, sensor technology is expanding our understanding of PD symptom burden, especially in motor and non-motor domains that have been challenging for providers to characterize and treat. Alongside this technological growth, the articles in this Research Topic demonstrate how leveraging objective data sets with machine learning and computing methods can impact symptom classification, symptom monitoring and treatment assessments, and treatment planning—creating a new multidisciplinary data-driven dimension of scientific inquiry and clinical care.

## Author contributions

All authors listed have made a substantial, direct, and intellectual contribution to the work and approved it for publication.
